# Determining concentric and eccentric force–velocity profiles during squatting

**DOI:** 10.1007/s00421-021-04875-2

**Published:** 2022-01-17

**Authors:** R. Armstrong, V. Baltzopoulos, C. Langan-Evans, D. Clark, J. Jarvis, C. Stewart, T. D. O’Brien

**Affiliations:** Research Institute for Sport and Exercise Sciences, Liverpool John Moores University, Tom Reilly Building, Byrom Street, Liverpool, L3 3AF UK

**Keywords:** Isovelocity, Multi-joint, In vivo, Assessment

## Abstract

**Purpose:**

The force–velocity relationship of muscular contraction has been extensively studied. However, previous research has focussed either on isolated muscle or single-joint movements, whereas human movement consists of multi-joint movements (e.g. squatting). Therefore, the purpose of this study was to investigate the force–velocity relationship of isovelocity squatting.

**Methods:**

Fifteen male participants (24 ± 2 years, 79.8 ± 9.1 kg, 177.5 ± 6 cm) performed isovelocity squats on a novel motorised isovelocity device (Kineo Training System) at three concentric (0.25, 0.5, and 0.75 m s^−1^) and three eccentric velocities (− 0.25, − 0.5, and − 0.75 m s^−1^). Peak vertical ground reaction forces, that occurred during the isovelocity phase, were collected using dual force plates (2000 Hz) (Kistler, Switzerland).

**Results:**

The group mean squat force–velocity profile conformed to the typical in vivo profile, with peak vertical ground reaction forces during eccentric squatting being 9.5 ± 19% greater than isometric (*P* = 0.037), and occurring between − 0.5 and − 0.75 m s^−1^. However, large inter-participant variability was identified (0.84–1.62 × isometric force), with some participants being unable to produce eccentric forces greater than isometric. Sub-group analyses could not identify differences between individuals who could/could not produce eccentric forces above isometric, although those who could not tended to be taller.

**Conclusions:**

These finding suggest that variability exists between participants in the ability to generate maximum eccentric forces during squatting, and the magnitude of eccentric increase above isometric cannot be predicted solely based on a concentric assessment. Therefore, an assessment of eccentric capabilities may be required prior to prescribing eccentric-specific resistance training.

## Introduction

The force–velocity (F–V) relationship defines an important dynamic property of muscle contraction (Alcazar et al. 2019; Fenn and Marsh 1935; Hill 1938). In isolated muscles, eccentric forces during lengthening of an active muscle are known to be up to 80% greater than isometric forces (Edman 1988). However, in vivo, where muscle forces are applied and measured as joint moments, the moment-velocity relationships display smaller and more variable differences between eccentric and isometric joint moments. The magnitude of this difference depends on the joints involved; for elbow flexion/extension 12–25% (Chapman et al. 2005; Hortobágyi and Katch 1990; Komi 1973), for ankle dorsi/plantar-flexion 12–18% (Connelly and Vandervoort 2000; Liederbach and Hiebert 1997), for knee extension 0–22% (Dudley et al. 1990; Melo et al. 2016; Pain and Forrester 2009), and for hip extension 8–11% (Boling et al. 2009).

The reduced eccentric enhancement of joint moments in vivo is thought to be due to a unique eccentric neural activation strategy (Enoka 1996) that decreases; voluntary activation (~ 15%) (Babault et al. 2001; Beltman et al. 2004), motor unit firing rate (~ 35%) (Del Valle and Thomas 2005), and cortical and spinal excitability (Duclay et al. 2011, 2014), when compared to isometric contractions. It is theorised that if it were not for these neural factors, the eccentric joint moment would be ~ 60% greater than typically observed (Pain and Forrester 2009). Due to these neural constraints and the variability of their effect, F–V relationships must be established in vivo so that the complexity of co-ordinating human movement may be considered, rather than relying on *ex-vivo* measurements, before eccentric loading recommendations for applied training can be made.

Our current understanding of the eccentric portion of the F*–*V relationship in vivo has primarily been derived from single-joint movements, e.g. hip extension (Boling et al. 2009), knee extension (Dudley et al. 1990; Melo et al. 2016; Pain and Forrester 2009), and plantar-flexion (Connelly and Vandervoort 2000; Liederbach and Hiebert 1997). Although single-joint models account for the neural constraints of voluntary contractions and are experimentally appealing as they allow tighter control of movement variables (e.g. joint/muscle/fibre velocity, angle/muscle length, and range of movement), human movement is not isolated into single-joints, but is rather a combination of multi-joint movement patterns. Due to the increased complexity of multi-joint and differing neural activation strategies (Behm et al. 2003) compared to single-joint movements, multi-joint F–V relationships are likely to differ from single-joint F–V relationships.

Studies of the multi-joint F–V relationship have demonstrated that the concentric portion of multi-joint F–V relationships, for example the rising phase of a loaded squat, are typically quasilinear (Bobbert 2012; Rahmani et al. 2001; Zivkovic et al. 2017). This is in contrast to single-joint F–V relationships, which are described as curvilinear (de Brito Fontana et al. 2014; Hauraix et al. 2017; Pain and Forrester 2009). These multi-joint F–V relationships have been performed in both traditional movements (e.g. squatting) (Spudić et al. 2020), and ballistic movements (e.g. sprinting, jumping, push-offs) (Morin and Samozino 2016; Samozino et al. 2010). This has resulted in practitioners being able to identify performance characteristics for improvement, that can then be targeted with training interventions, based upon the slope of the concentric F–V curve compared to a calculated optimal profile (Samozino et al. 2012).

Unfortunately, the majority of the multi-joint F–V research has focussed on the concentric portion (Spudić et al. 2020), and much less evidence exists regarding the nature of the eccentric portion of the multi-joint force–velocity relationship in vivo, likely due to the difficulty, and inherent risk, of applying supra-maximal external loads during high movement velocities. To our knowledge, there is only one study to date which has investigated the eccentric portion of the F–V relationship in a lower body multi-joint task (Hahn et al. 2014). Utilising a leg-press model, eccentric ground reaction forces were up to 15% greater than isometric forces. Eccentric force production peaked at a knee flexion velocity of − 60°/s and decreased as eccentric velocity increased to − 180°/s (Hahn et al. 2014). This suggests that the eccentric portion of multi-joint F–V relationship is similar in shape to the single-joint F–V relationship, albeit with a reduced eccentric enhancement. However, the leg-press as used by Hahn et al. (2014) does not allow for full hip extension, and is not as effective at improving athletic performance qualities as the squat (Wirth et al. 2016). Although previous studies have examined the force characteristics of the eccentric phase of the squat (McNeill et al. 2021; Frohm et al. 2007), to our knowledge, no previous study has investigated the eccentric portion of the F–V relationship in the squat.

**Fig. 1:**
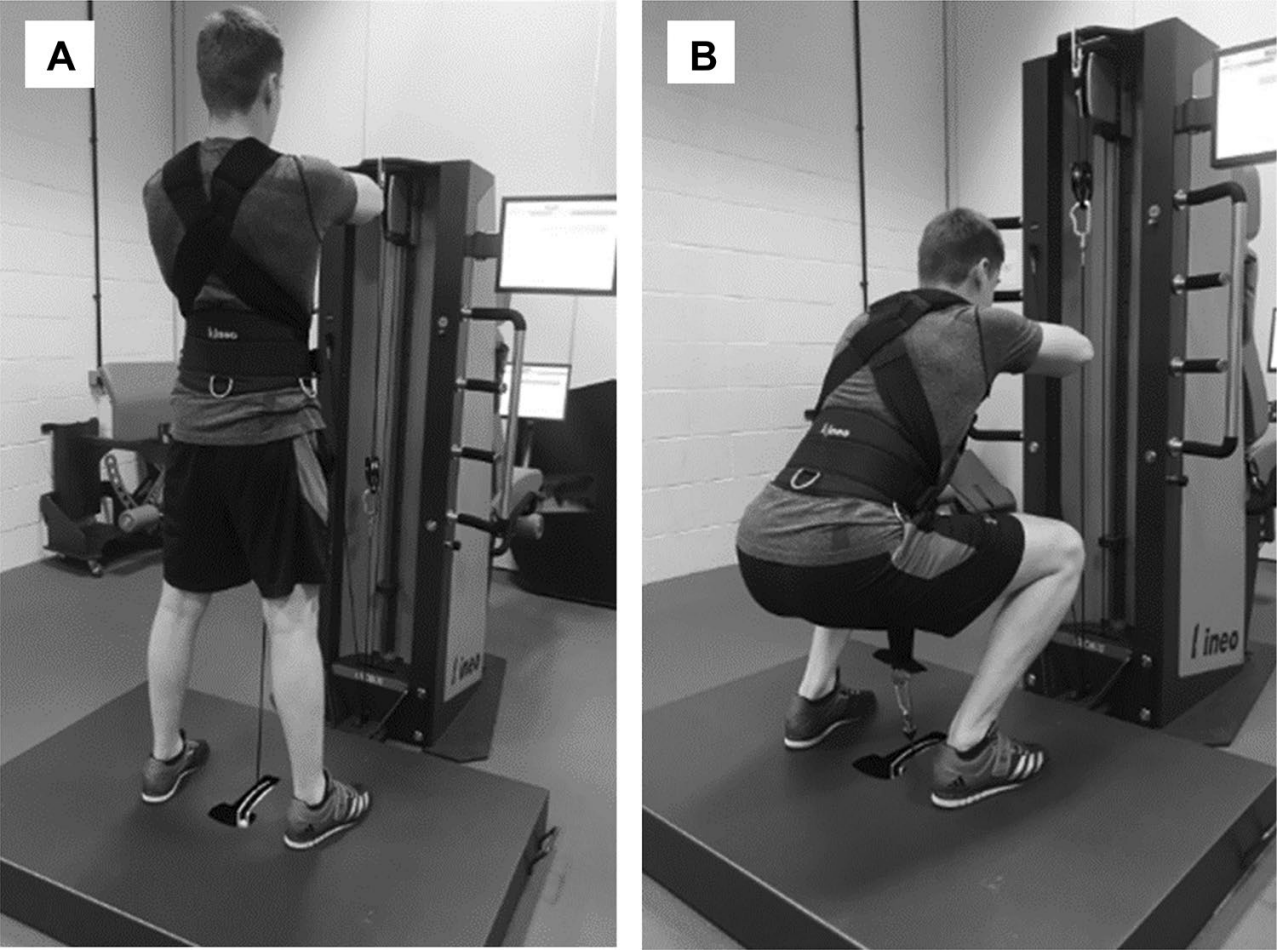
Kineo Training System; participant is connected to an electric motor via a hip/shoulder harness attached to a cable pulley system. **A** The start of the eccentric phase/end of concentric phase, **B** the end of the eccentric phase/start of the concentric phase. Two additional force plates, one under each foot, where added to this experimental setup (not shown) to measure vertical ground reaction forces (N)

Studying the F–V profile of squatting is complicated because the muscular effort required to control the speed of descent of any given load increases throughout the eccentric phase as the hips and knees flex (Bryanton et al. 2012). Although an individual may be able to withstand a supra-maximal load at the start of a squat, there is an increased likelihood of failure, concomitant with risk of injury, during the approach to a deeper squat position. Furthermore, movement velocity varies over the duration of the movement (Miletello et al. 2009), so accurately measuring eccentric force and velocity over repeated trials may prove challenging. To overcome these difficulties, advances in technology, using the Kineo Training System (v7.0, Globus, Italy) (Fig. 1), allow for the application of multi-joint isovelocity movements, by manipulating the external force at a constant velocity over the duration of the exercise. This would, therefore, allow concentric and eccentric isovelocity squatting to occur in a safe, controlled manner whilst collecting ground reaction forces, thus overcoming the limitations of previous eccentric loading approaches.

Therefore, the primary aim of this study was to establish the complete F–V relationship during isovelocity squatting. This knowledge will allow the development of evidence-based training recommendations for future eccentric overload interventions. In current practice, accentuated eccentric training loads (AEL) are typically up to 20% greater than the concentric one-repetition max (Harden et al. 2020). However, this relies on the assumption that this overload is suitable for all individuals which may not be correct as the maximum eccentric strength of individuals may vary. Therefore, a secondary aim of this study was to identify whether concentric strength influences the magnitude of the eccentric force increases above the isometric level. It was hypothesised that (1) eccentric squatting forces would be greater than isometric, but less than the 30% above isometric force common in single-joint eccentric contractions; and (2) that the ability to produce eccentric squatting forces above isometric would be associated with squatting ability, as reflected by performance in conventional concentric squatting.

## Methods

### Participants

Fifteen resistance trained males (age 24 ± 2 years, body mass 79.8 ± 9.1 kg, height 177.5 ± 6 cm, training age 3.5 ± 1.5 years) volunteered for this study. All participants could demonstrate a good squatting technique, as determined by a qualified strength and conditioning coach, and frequently (> 1 × per week) performed the squat (or variation) within their habitual resistance training practice. Resistance-trained participants were selected for this study to limit the known negative effects of the eccentric neural activation strategy in untrained participants (Aagaard 2018). Prior to participation, written informed consent was completed and this study received ethical approval from Liverpool John Moores University research ethics committee (19/SPS/038).

### Experimental protocol

Participants reported to the Liverpool John Moores laboratories on three occasions. The first and second visits were used for participant familiarisation with the experimental protocols, and to measure body mass (to the nearest 0.1 kg, on electronic scales; SECA, Germany), and height (to the nearest 0.5 cm, with a stadiometer; SECA, Germany). Participants completed a standardised warmup following the RAMP protocol (Jeffreys 2006), which was concluded with several progressively heavier isotonic squats on the Kineo Training System on which all experimental trials were also completed. Following the warmup, participants underwent a familiarisation session inclusive of concentric and eccentric isovelocity squatting. Squat stance was standardised with feet shoulder-width apart and externally rotated ~ 20°. Squatting range of motion was determined, whereby the eccentric phase started with the participant standing with hips and knees fully extended and lasted until the participant had squatted down to a depth where the top of the thigh was parallel to the ground. The concentric phase began after the eccentric phase finished and until the participant had fully extended the hips and knees (Fig. 1). Squatting depth was confirmed by analysis of cable displacement during experimental trials. Pilot data (*n* = 4) from our laboratory identified that following two familiarisation sessions, forces produced during the − 0.5 m s^−1^ eccentric trail produced a variation of 1.9%, this is in line with previous research and recommendations (Hahn 2018; McMaster et al. 2014), and therefore, sufficient familiarisation was achieved.

Experimental data were collected during the third visit, 4–10 days following the last familiarisation session. Participants refrained from strenuous physical activity for 48 h prior to testing and were asked to arrive in a fed and hydrated state. Following the standardised warmup, participants completed a total of six maximum effort isovelocity trials at 0.75, 0.5, 0.25, − 0.25, − 0.5, and − 0.75 m s^−1^, whereby positive and negative values were indicative of concentric and eccentric directions, respectively, with three repetitions per trial.

**Fig. 2:**
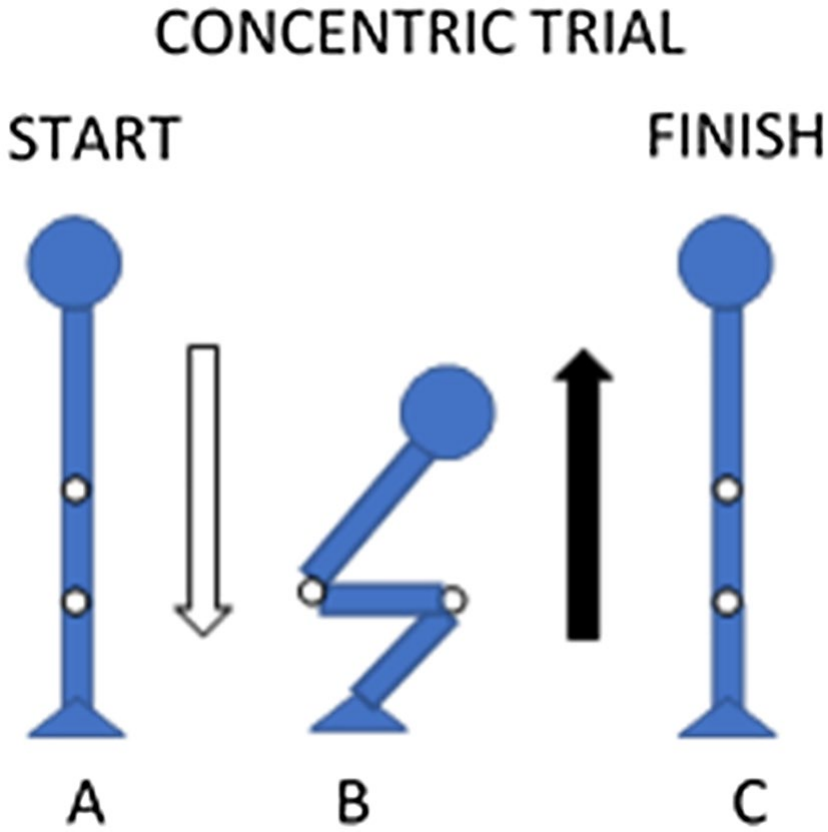
Schematic of concentric isovelocity squatting trials. **A** Start position, **B** submaximal eccentric squat to parallel squat depth, and **C** maximal effort concentric squat. Arrows represents direction of movement, solid-black arrow denotes maximum effort trial that is recorded for data analysis

During concentric trials, participants began by standing with the hips and knees extended, performed a submaximal (~ 80% perceived effort) eccentric isovelocity squat at − 0.25 m s^−1^, immediately followed by a maximum effort isovelocity concentric squat at the prescribed trial velocity (Fig. 2). Participants were provided visual feedback to ensure they produced an effort of 80% during the submaximal eccentric phase, with this value having been identified during the second familiarisation session.

**Fig. 3:**
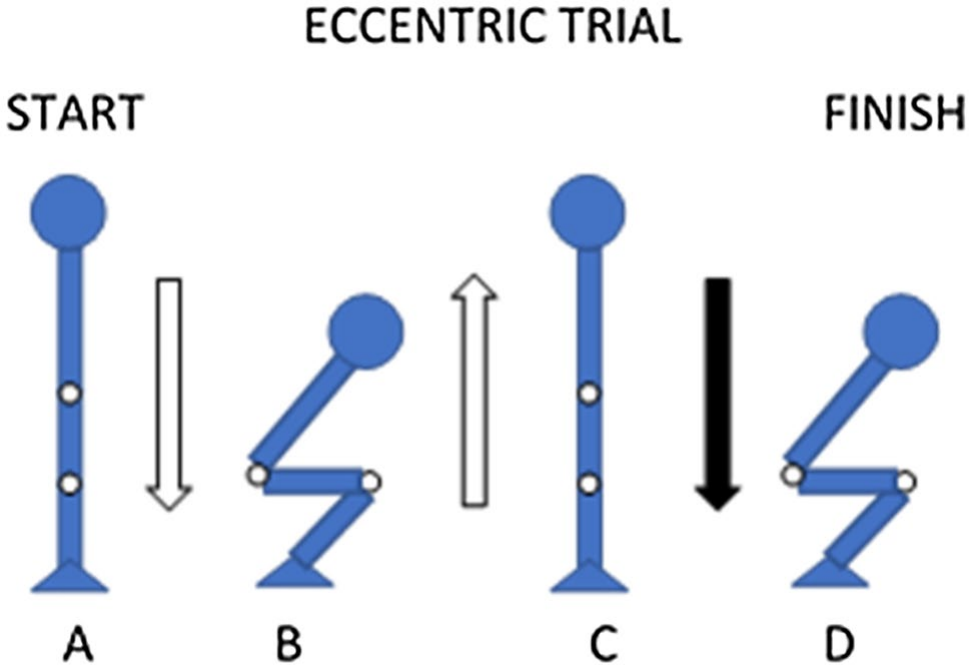
Schematic of eccentric isovelocity squatting trials. **A** Start position, **B** submaximal eccentric squat to parallel squat depth, **C** near-maximal concentric squat to full hip/knee extension to preload, and **D** maximal effort eccentric squat. Arrows represents direction of movement, solid-black arrow denotes maximum effort trial that is recorded for data analysis

During eccentric trials, participants began by standing with the hips and knees extended, performed a submaximal eccentric isovelocity squat (~ 80% perceived effort, − 0.25 m s^−1^), followed by a near-maximal concentric isovelocity squat (~ 90% perceived effort, 0.25 m s^−1^), before performing the maximal effort isovelocity eccentric squat for which data were recorded (Fig. 3). Visual feedback was provided as per the concentric trials. The near-maximal concentric effort immediately prior to the maximal eccentric effort ensured preload on the musculature, which is required for maximal eccentric efforts (Hahn 2018; Linnamo et al. 2006). During the maximal eccentric trial, the participant maximally resisted the downwards displacement of the external cable at the respective velocity until the end of the range of motion. Three repetitions were completed at each velocity, with 5-min passive rest between each trial.

### Data acquisition and analyses

During all trials, ground reaction forces (N) under each foot were collected via a dual force plate system (9287c, Kistler, Switzerland), sampling at 2000 Hz. Analogue signals were amplified and converted to a digital signal prior to being collected in Qualisys Track Manager (Qualisys, Sweden) and then exported to Visual 3D (C-Motion, USA) for subsequent analysis. The greatest peak vertical ground reaction forces from each of the six experimental conditions (Concentric; 0.75, 0.5, and 0.25 m s^−1^, Eccentric; − 0.25, − 0.5, & − 0.75 m s^−1^) were used for analysis. Ground reaction forces were then processed via a fourth-order Butterworth filter with a cutoff frequency of 6 Hz; then, the forces of the dominant and non-dominant limb were summed together.

During these trials, only forces that occurred during the isovelocity phase of the squat were used, which were defined from the measured movement velocity profile. To confirm actual squat velocity for each defined trial, reflective markers were placed on the cables which attached the participant to the Kineo Training System, and monitored by three 3D motion capture cameras (Opus 3 series, Qualisys, Sweden), sampling at 200 Hz.

Forces were plotted against the target velocity to create F–V relationships for each participant. Forces were normalised against a predicted isometric force. A joint-angle specific maximum isometric force could not be measured as peak forces occur at different joint angles during the concentric and eccentric phase (Melo et al. 2016), and between participants. Instead, isometric force was calculated for zero velocity from a cubic polynomial regression equation fitted to each participant’s measured force–velocity profile. Calculating isometric force in this manner has been previously used (Morin and Samozino 2016; Samozino et al. 2010) and shown to be robust.

### Statistical analyses

All data were statistically analysed using SPSS (version 26, IBM, USA). A one-way repeated measures ANOVA with six factor levels was used to test for differences in the peak force from each velocity. As there was a violation of sphericity *(P* < 0.001), a Greenhouse–Geisser correction was used (Atkinson 2001). A two-way repeated measures ANOVA (2 × 6) was used to test for differences between the dominant and non-dominant limbs. Finally, a one-way repeated measures ANOVA was used to tests for differences in the squat depth (%) at which peak force occurred (whereby 0% is the position at which the hips and knees are fully extended, and 100% is the position at which the thighs were parallel to the ground). Significance was accepted when *P* < 0.05. Statistically significant results underwent a Holm–Bonferroni post hoc analysis. All data are presented as mean ± SD, unless otherwise stated. Correlation analysis was performed to determine if maximal concentric strength influenced eccentric force production. Absolute and normalised peak force from all eccentric trials (− 0.25, − 0.5, and − 0.75 m s^−1^) were correlated against the trial in which the greatest concentric force was produced (0.25 m s^−1^).

Coefficients of variation and intraclass correlation coefficients were performed to identify the reliability of ground reaction forces between repetitions at each velocity. Intraclass correlation coefficient was interpreted in line with recent guidelines (Koo and Li 2016). Lastly, Bland–Altman analyses (Bland and Altman 1986) and limits of agreement (LOA) were used to identify if the measured velocity differed from the target velocity.

## Results

**Fig. 4:**
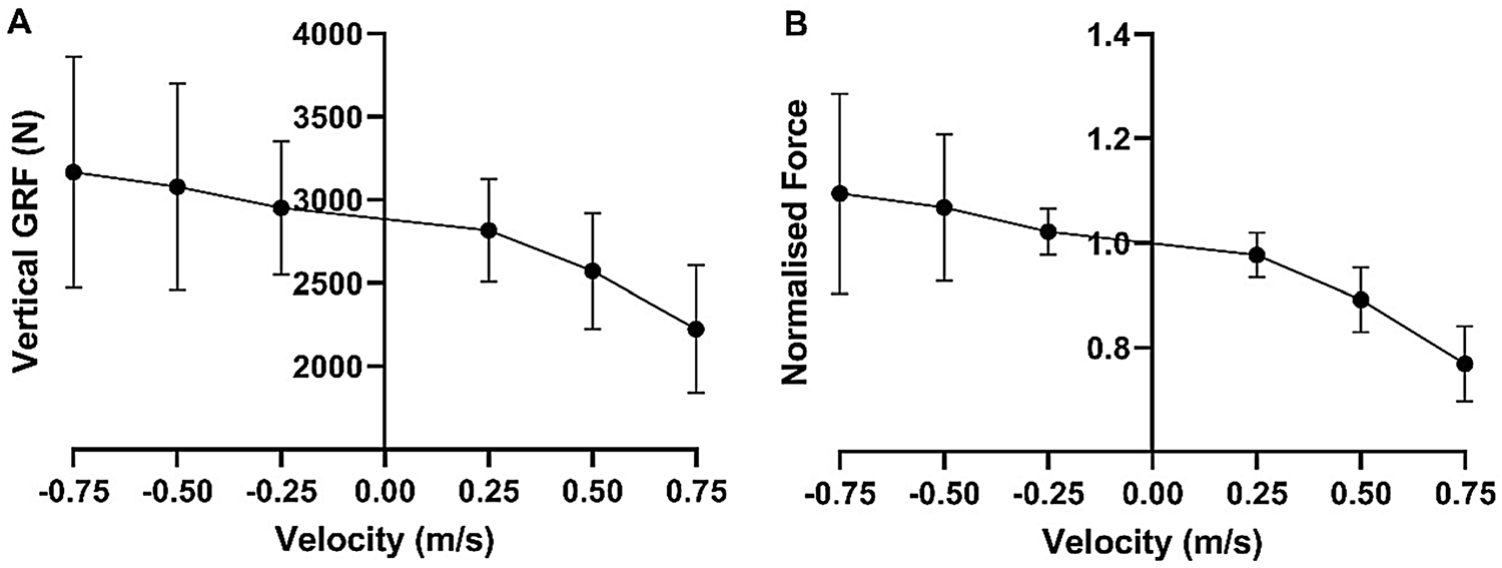
Group mean ± SD force–velocity relationships of isovelocity squatting. **A** Vertical ground reaction force (N). **B** Normalised force relative to isometric. Concentric velocities are +ve, eccentric velocities are -ve

The group mean force during isovelocity squatting conformed to the expected in vivo F–V profile, with the maximum force 1.095 times greater than isometric, which was recorded during the highest velocity eccentric trial (− 0.75 m s^−1^) (Fig. 4). There was a significant main effect of squat velocity on vertical ground reaction force (F_1.85, 25.87_ = 22.059, *P* < 0.001).

Post hoc analysis identified that the eccentric − 0.75 m s^−1^ velocity trial (*P* = 0.037, 95% CI of Δ = 24 to 657 N) and the − 0.5 m s^−1^ velocity trial (*P* = 0.037, 95% CI of Δ = 18 to 509 N) both produced greater mean peak forces than the highest recorded concentric velocity trial (0.25 m s^−1^). However, the difference in the peak force between the eccentric − 0.25 m s^−1^ and concentric 0.25 m s^−1^ trials did not reach significance (*P* = 0.288, 95% CI of Δ = − 14 to 287 N), nor between the eccentric − 0.75 and − 0.5 m s^−1^ trials (*P* = 0.300, 95% CI of Δ = − 86 to 258 N). Peak forces occurred at 36–41% (± 6–14%) of squat depth regardless of squat direction and velocity (F_5_ = 0.846, *P* = 0.521).

There was an asymmetry in the forces produced between the dominant and non-dominant limbs (F_15_ = 10.002, *P* = 0.007), with the smallest limb asymmetries identified during the higher velocities (~ 3%), and largest asymmetries occurring during the slow velocities (~ 6%). However, there was not a significant interaction of the dominant vs non-dominant limb on the magnitude of forces produced at each velocity (F_5_ = 0.522, *P* = 0.759), and thus did not change the shape of the F–V relationship.

### Individual responses

**Fig. 5:**
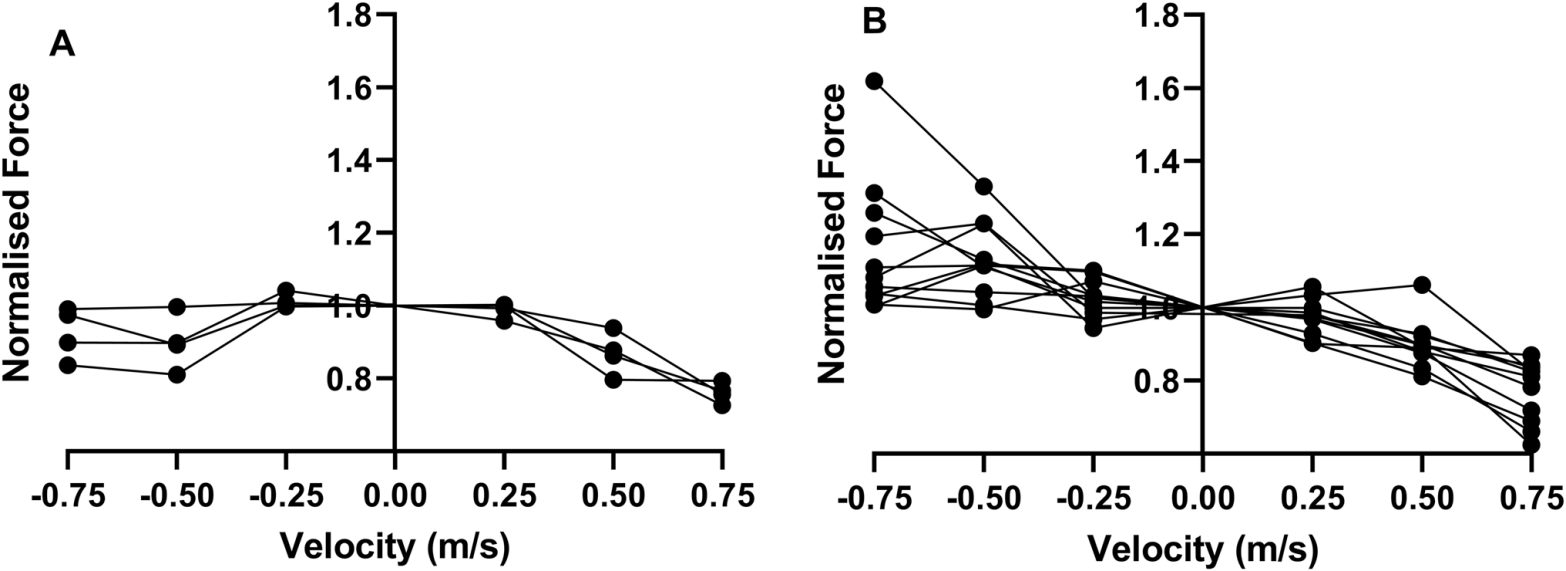
Force–velocity relationship from isovelocity squatting in **A** sub-group of participants that did not achieve an eccentric force increase (normalised eccentric force ≤ 1.0) (*n* = 4) and **B** sub-group of participants that did achieve an eccentric force increase group (normalised eccentric force > 1.0) (*n* = 11)

There was large inter-participant variability between the eccentric forces produced. Analyses of individual data revealed that some participants did not produce eccentric forces greater than isometric (Fig. 5). Table 1 summarises the characteristic differences between those individuals who had no eccentric increase (normalised eccentric force ≤ 1.0 across all trials) and those who had an eccentric increase (> 1.0). No significant differences were found between groups (*P* = 0.059–0.971), although the no eccentric-increase group tended to be taller and heavier.

**Table 1:** Individual and means ± SD for characteristics of participants who did not achieve an eccentric increase in force (*n* = 4) and those who did (*n* = 11)

	Participant	Normalised eccentric force (− 0.75 m s^−1^)	Body mass (kg)	Height (cm)	Age (years)	Barbell squat 1RM (kg)	Training age (years)	Squat 1RM/BM
No eccentric increase	k6	0.84	85.65	193.5	26	120	3	1.40
	k5	0.90	83.6	179.5	22	150	2	1.79
	k4	0.97	82.85	182	25	120	3	1.45
	k9	0.99	94	173	25	200	7	2.13
Mean ± SD		0.93 ± 0.07	86.5 ± 5	182 ± 8.5	25 ± 2	147.5 ± 37.5	4 ± 2	1.69 ± 0.33
Eccentric increase	k12	1.00	80.7	182	24	127.5	4	1.58
	k2	1.01	69	178.5	23	105	5	1.52
	k3	1.03	68.6	171	20	125	3	1.82
	k8	1.04	63	172	24	92.5	1	1.47
	k11	1.06	81.3	178.5	23	150	4	1.85
	k10	1.08	87.4	175	23	140	5	1.60
	k1	1.11	65.5	174	24	115	4	1.76
	k7	1.19	88.3	178.5	27	140	2	1.59
	k14	1.26	82.7	172	21	132.5	4	1.60
	k15	1.31	84.1	181.5	27	140	4	1.66
	k13	1.62	81	171	26	120	2	1.48
Mean ± SD		1.16 ± 0.18	77 ± 9.5	175 ± 4	24 ± 2	126 ± 18	3 ± 2	1.63 ± 0.13

Data are in ascending rank order for normalised maximum eccentric force

**Fig. 6:**
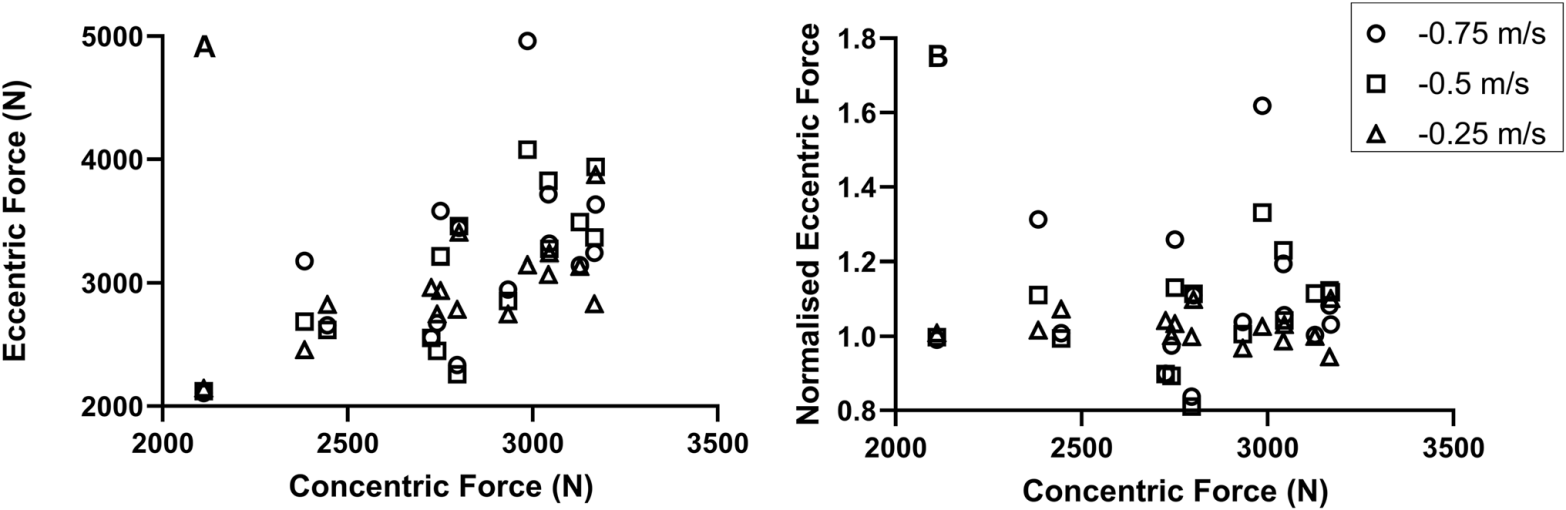
Scatter plots showing **A** positive linear correlation between concentric force and eccentric force at − 0.75 (*P* = 0.036), − 0.5 (*P* = 0.001), & − 0.25 m s^−1^ (*P* = 0.002). **B** No correlation between concentric force and normalised eccentric force (*P* = 0.19–0.757)

In addition, there was a modest to high positive correlation between absolute peak concentric force (0.25 m s^−1^) and absolute peak eccentric force (− 0.75 m s^−1^; *r*_*15*_ = 0.544, 95% CI of Δ = 0.04 to 0.83, *P* = 0.036, − 0.5 m s^−1^; *r*_*15*_ = 0.745, 95% CI of Δ = 0.38 to 0.91, *P* = 0.001, − 0.25 m s^−1^; *r*_*15*_ = 0.738, 95% CI of Δ = 0.36–0.91, *P* = 0.002) (Fig. 6A). However, there was no significant correlation between absolute peak concentric force (0.25 m s^−1^) and the isometric-normalised eccentric force (− 0.75 m s^−1^; *P* = 0.757, − 0.5 m s^−1^; *P* = 0.19, − 0.25 m s^−1^; *P* = 0.628) (Fig. 6B).

Analysis of repetition-to-repetition variation of vertical ground reaction forces within each velocity identified acceptable coefficients of variation (6.1–9.2%) and intraclass correlation coefficients (0.84–0.93) (McMaster et al. 2014). These values are similar to those reported for both traditional (Fairus et al. 2016), and isometric squats (Palmer et al. 2018). Finally, measured cable velocity was, on average ~ 0.02 m s^−1^ greater than that of each target velocity (*P* < 0.001, LOA = 0.002:0.037) [exact velocities (and percentage difference) are as follows; − 0.767 m s^−1^ (2.2%), − 0.519 m s^−1^ (3.7%), − 0.261 m s^−1^ (4.3%), 0.268 m s^−1^ (6.9%), 0.522 m s^−1^ (4.3%), and 0.769 m s^−1^ (2.5%)]. Therefore, the conclusions would be the same if we had used measured velocity rather than target velocity in our calculations.

## Discussion

The main findings of this study establish that maximal isovelocity squatting conforms to the well-established pattern of the force–velocity relationship, with peak eccentric forces being ~ 10% greater than isometric forces. However, large inter-participant variability existed at higher eccentric velocities. Although most participant conformed to the expected F–V profile, some individuals did not produce eccentric forces greater than isometric, whilst one produced an extremely high eccentric force (Table 1, participant K13).

We accept the first hypothesis since the group mean eccentric force peak was ~ 10% greater than isometric (Fig. 4), but this is much smaller than the 30% difference previously reported in single-joint F–V relationships (Alcazar et al. 2019). These values are both far below isolated muscle forces which can reach up to 80% greater than isometric (Edman 1988). These differences are likely explained by altered activation levels occurring during multi-joint movements compared to single-joint movements (Behm et al. 2003), which may impair the ability to produce maximal force (Maffiuletti et al. 2016). In addition, the greater the degrees of freedom within a movement, the more unstable a joint becomes (Wuebbenhorst and Zschorlich 2011), requiring the musculature to stabilise the movement rather than produce maximal force (Kornecki and Zschorlich 1994; Wuebbenhorst and Zschorlich 2012). These two neural mechanisms could cause a general decrease in force production, which is consistent with previous literature (Bryanton et al. 2012) showing that during concentric squatting, the lower body musculature can only produce 60–80% of its predicted maximum force compared to when tested in a single-joint isometric state. Rapid increases in neural activation levels have been shown during eccentric-specific resistance training (i.e. modulation of the load/velocity of the eccentric phase of an exercise) (Seynnes et al. 2007), therefore, it may be hypothesised that rapid improvements in eccentric squatting strength may be achieved by overcoming the neural limitations of eccentric squatting following a short-term training intervention.

In addition, unlike single-joint movements where kinematics are constrained, the kinematics of squatting can differ between the concentric and eccentric phase (Swinton et al. 2012), which may prevent the hip and knee joints simultaneously being at their optimal angle to produce maximal joint moments, despite squat depth remaining constant. The combined contribution of the two joints to the ground reaction force could, therefore, be reduced (Beckham et al. 2018), in particular during the eccentric trials compared to the concentric trials, reducing the eccentric squatting force. Future studies should utilise inverse dynamics to study the individual joint contributions to eccentric squatting, and assess squatting kinematics, rather than just squat depth, to better understand the mechanisms contributing to the strength capacity during eccentric squatting and inform targeted training prescription guidance.

The F–V relationship of squatting followed the same sigmoidal shape that exists in single-joint actions and isolated muscle (Alcazar et al. 2019), reflecting the established mechanics of muscle contraction. The shape of the force–velocity curves produced in this study were also similar between the dominant and non-dominant limbs, with the asymmetries between limbs (< 6%) being similar to the asymmetries previously reported in bilateral movements (Simon and Ferris 2008). However, following the initial increase in eccentric force from − 0.25 m s^−1^ to − 0.5 m s^−1^, there was a plateau between − 0.5 m s^−1^ and − 0.75 m s^−1^. In practical terms, it appears that there exists an optimal velocity range that facilitates the greatest production of eccentric forces, which in turn should produce the greatest physiological response (Rindom et al. 2019). Our data suggest that the greatest forces occur between − 0.5 and − 0.75 m s^−1^. However, due to the individual differences when performing eccentric actions (discussed below), it may be prudent to perform assessments of eccentric capabilities prior to prescribing eccentric resistance training protocols.

### Individual differences

When exploring our data, it becomes evident that a greater variation existed amongst the eccentric trials than amongst the concentric trials (see the standard deviations in Fig. 4). At − 0.75 m s^−1^, the normalised force ranged from 0.84 to 1.62 around a mean of 1.1, indicating that although many individuals generated the physiologically expected eccentric force above isometric (Fig. 5B), some did not (Fig. 5A), even though all participants were familiar with resistance training and the squat. This large inter-participant variability was still apparent even when excluding the participant who achieved 1.6 × isometric. Variability between individuals in the ability to produce eccentric moments has been reported previously during knee extension/flexion (Hahn 2018). Group mean knee moments were reported as 1.2 × isometric, however, some individuals were shown to be capable of producing moments 1.8 × isometric (Hahn 2018). Therefore, our measurement of a maximal eccentric force of 1.6 × the isometric agrees with the limited previous data.

We assessed whether the individual ability to generate eccentric forces was associated with overall squatting ability. However, the normalised eccentric force was not correlated with absolute concentric force (Fig. 6B), and so we find that strength itself did not determine whether an individual produced eccentric forces greater or less than isometric. This is supported by previous research that has shown that we cannot accurately predict eccentric strength from a concentric strength test (Harden et al. 2019). Therefore, we reject hypothesis 2. Previous training interventions have demonstrated that eccentric-specific resistance training causes an increase in eccentric force production (Seger et al. 1998; Spurway et al. 2000), probably due to movement-specific improvements in muscle activation and a greater lengthening of muscle fascicles (Franchi et al. 2014). Therefore, we would expect individuals with a history of eccentric-specific resistance training to display a greater normalised eccentric force. However, although all participants had a history of resistance training (3.5 ± 1.5 years) and could squat a minimum of 1.4 × body weight (Table 1), none of these participants had a notable history of eccentric-specific resistance training, so this does not explain the variances found in this study.

There are other factors that may influence eccentric squatting force that this study did not specifically assess, but comparison of the sub-groups (Table 1) may offer some insight. Although all participants squatted to a depth where the centre of the hip was below the centre of the knee, squatting technique varies between individuals (Myer et al. 2014). Although not statistically significant, the group that had no eccentric increase was taller. Taller individuals may adopt a more hip-dominant squatting technique to counter balance (Myer et al. 2014), and this technique can change between the concentric and eccentric phase. This may result in differing hip, knee, and ankle joint ranges of motion, which would influence the amount of force exerted into the ground (Beckham et al. 2018), and thus the profile of the F–V relationship. Future research may explore the effects of height and limb lengths on the joint contributions and their effect on exploiting the greater strength capacity of eccentric squatting.

Another factor worth considering is the ability to activate the musculature during eccentric squatting. This could explain the large inter-participant variability. Although it was not assessed in this study, the ability to activate skeletal muscle has been shown to correlate with force production (Folland et al. 2014). Eccentric contractions have a unique neurological activation, compared to concentric/isometric (Enoka 1996) and activation capacity is known to differ between individuals (Avrillon et al. 2021), furthermore eccentric activation can be trained (Aagaard et al. 2000). Therefore, measurements of eccentric activation should be included in future research.

Regardless of the reasons why certain individuals were able to produce more or less eccentric normalised force, practitioners and researchers need to be aware that this variability is real and of a large magnitude, and future research on eccentric-specific resistance training should take this into consideration.

### Practical applications

Our data suggest that maximal concentric strength does not influence the ability to maximise relative eccentric force production, and therefore practitioners should attempt to measure eccentric capability prior to prescribing eccentric-specific resistance training rather than relying on standardised loads relative to concentric maximums (Harden et al. 2020). This lack eccentric-specific assessment, and thus individualisation of training programs, may explain why the efficacy of eccentric-specific resistance training (e.g. accentuated eccentric loading) has been debated in the past (Douglas et al. 2018).

In many applied settings, eccentric-specific assessment is achieved under external load (Harden et al. 2019), which will dictate movement velocity, rather than the imposition of isovelocity movements. This presents practical challenges if done using traditional weightlifting techniques. In this study, however, the Kineo training system proved effective in delivering the fast eccentric squatting efforts required to identify a plateau in eccentric force, allowing individualised F–V profiles to be developed. However, for practitioners that do not have access to this equipment, field-based assessment of eccentric capabilities may need to be developed to individualise eccentric-specific resistance training. Future research should also examine the effects of different eccentric protocols on movement velocity, and subsequent force production, and the ability of resistance training interventions to train and improve these aspects of performance.

Although this study focussed on establishing underpinning knowledge of the multi-joint force velocity curve, some findings may be extrapolated to applied practice. In applied settings, AEL is often coupled with a slower eccentric velocity, which results in an eccentric phase duration of 3–4 s (Harden et al. 2020), equivalent to a velocity slower than − 0.25 m s^−1^. However, our data suggest that at this slow eccentric velocity, AEL squatting may only provide a 2% benefit in terms of peak forces imposed on the body, when compared to maximal effort traditional squatting. In contrast, the data reported here demonstrate that many trained individuals are able to generate larger forces and experience greater training loads in faster eccentric trials, which may provide an accentuated stimulus for adaptation. However, future studies will need to be performed to confirm this.

Lastly, there are some limitations to this study. First, this study did not assess the kinematics of squatting, and as such, we do not know the joint angles at which peak ground reaction force occurred, nor do we know if taller individuals adopted a different squatting technique (as proposed in the individual differences section). An analysis of squatting kinematics could solve these limitations, and therefore, should be included in future research. Second, this study only assessed three eccentric velocities, and found that peak eccentric force occurred between − 0.5 and − 0.75 m s^−1^. Therefore, future research should examine the effects of a greater range of velocities (targeted at the faster velocities) on ground reaction forces, and attempt to identify the participant characteristics that determine individual differences.

## Conclusion

The main finding from this investigation is that the isovelocity squatting F–V relationship conforms to the typical in vivo F–V profile with eccentric forces greater than isometric. However, the group mean normalised eccentric forces (1.1 × isometric) were lower than those typically reported in single-joint contractions (1.3 × isometric). Large inter-participant variability existed in the eccentric forces produced, with some participants producing eccentric forces up to 1.62 × isometric, but others half of that and not exceeding isometric (0.84 × isometric). Concentric strength and training age did not appear to determine the ability to maximise eccentric force production. Our data suggest that higher eccentric velocities result in greater force production, therefore, practitioners may wish to select AEL protocols that permit safe application of a velocity of ~ − 0.5 m s^−1^, or an eccentric tempo of ~ 1 s, if maximising eccentric force production is the objective of a training session. However, an assessment of eccentric capabilities is important to individualise training interventions, owing to the large inter-participant variability in eccentric force production (Fig. 6).

## Data Availability

The datasets generated during the current study are available from the corresponding author on reasonable request.

## Abbreviations

AEL: Accentuated eccentric loading
ANOVA: Analysis of variation
F–V: Force–velocity
LOA: Limits of agreement
